# *Taxus wallichiana* var. *chinensis* (Pilg.) Florin Aqueous Extract Suppresses the Proliferation and Metastasis in Lung Carcinoma *via* JAK/STAT3 Signaling Pathway

**DOI:** 10.3389/fphar.2021.736442

**Published:** 2021-11-16

**Authors:** Leitao Sun, Shuning Ding, Qi Luo, Peipei Wang, Xiao Yang, Linqin Wu, Yangfan Chen, Xueer Zheng, Hang Zhang, Li Yuan, Shanming Ruan, Changsheng Xie

**Affiliations:** ^1^ Department of Medical Oncology, The First Affiliated Hospital of Zhejiang Chinese Medical University (Zhejiang Provincial Hospital of Traditional Chinese Medicine), Hangzhou, China; ^2^ The First School of Clinical Medicine, Zhejiang Chinese Medical University, Hangzhou, China; ^3^ Qingbo Community Health Service Center of Shangcheng District, Hangzhou, China

**Keywords:** lung cancer, proliferation, metastasis, JAK/STAT3 signaling pathway, *Taxus wallichiana* var. *chinensis* (Pilg.) Florin

## Abstract

As one of the most common neoplasms globally, lung cancer (LC) is the leading cause of cancer-related mortality. Recurrence and metastasis negatively influencing therapeutic efficacy and overall survival demand new strategies in LC treatment. The advantages of TCM are increasingly highlighted. In this study, we obtained the major chemical components and their ratios in the aqueous extract of *Taxus wallichiana* var. *chinensis* (Pilg.) Florin (AETW) by UPLC-Q/TOF-MS/MS detection. The CCK-8 assay revealed that AETW could selectively inhibit the growth of A549 and HCC827 cells in a dose-dependent manner with little effect on normal human lung cells. Moreover, both *in vitro* and *in vivo* experiments showed that AETW was able to suppress the capacities of cell migration and invasion and downregulate the EMT and the JAK/STAT3 signaling pathway. To further probe into the molecular mechanism, the overexpression of STAT3 was performed into LC cells with AETW treatment, which counteracted the inhibitory effect on malignant behaviors of A549 and HCC827 cells with the decline in the expressions of p-JAK and p-STAT3. Taken together, we propose that AETW may inhibit the proliferation and metastasis by inactivating the JAK/STAT3 axis.

## Introduction

Lung cancer (LC) remains a global public health problem characterized by its strong invasiveness and rapid metastasis (Ashrafizadeh et al., 2021a). Despite improvements made in the diagnosis, surgical techniques, health management, and adjuvant therapy in recent years, LC is closely related to high mortality due to its malignancy and invasiveness (Ashrafizadeh et al., 2021b). According to the American Cancer Society, there were 228,820 new cases and 135,720 deaths of LC in 2020, and LC ranked the first among malignancies (Miller et al., 2020). Furthermore, invasion and metastasis along with unlimited reproduction of LC cells induce poor response or even resistance to routine clinical strategies, further decreasing the survival (Abadi et al., 2021; Pacini et al., 2021). Therefore, searching for effective therapeutic strategies and targets is urgent and essential. It is worth mentioning that as an important source for the development of novel anti-tumor drugs, traditional Chinese medicine (TCM) is featured by the advantages of multiple targets, high efficacy, low toxicity, and low medical cost (Wang et al., 2021).

*Taxus wallichiana* var. *chinensis* (Pilg.) Florin (TW) is a natural anti-tumor plant distributed in the Yangtze River and its south area, which has been classified as endangered according to the IUCN Red List Criteria (Forest et al., 2018). According to the pharmacology of TCM, the flavor of TW is sweet and slightly bitter, and its nature is even. TW is favorable to the lung, stomach, and large intestine channels, which provides the theoretical foundation of TCM for its clinical use in lung carcinoma. Until the extraction of Taxol with a broad-spectrum anti-tumor effect from *Taxus* L. plant in the 1970s, TW has been widely concerned for its pharmacological value (Wani et al., 1971). Currently, abundant evidence has confirmed that the extract of TW is able to inhibit proliferation, induce apoptosis, strengthen immunity, and reverse drug resistance in lung cancer cells (Jiang et al., 2016; Zhang et al., 2021a). However, the function and the underlying mechanism of TW in preventing or reversing cancer metastasis have been rarely reported. Meanwhile, the specific chemical content in the aqueous extract of *Taxus wallichiana* var. *chinensis* (Pilg.) Florin (AETW) has been poorly understood.

**FIGURE 1:**
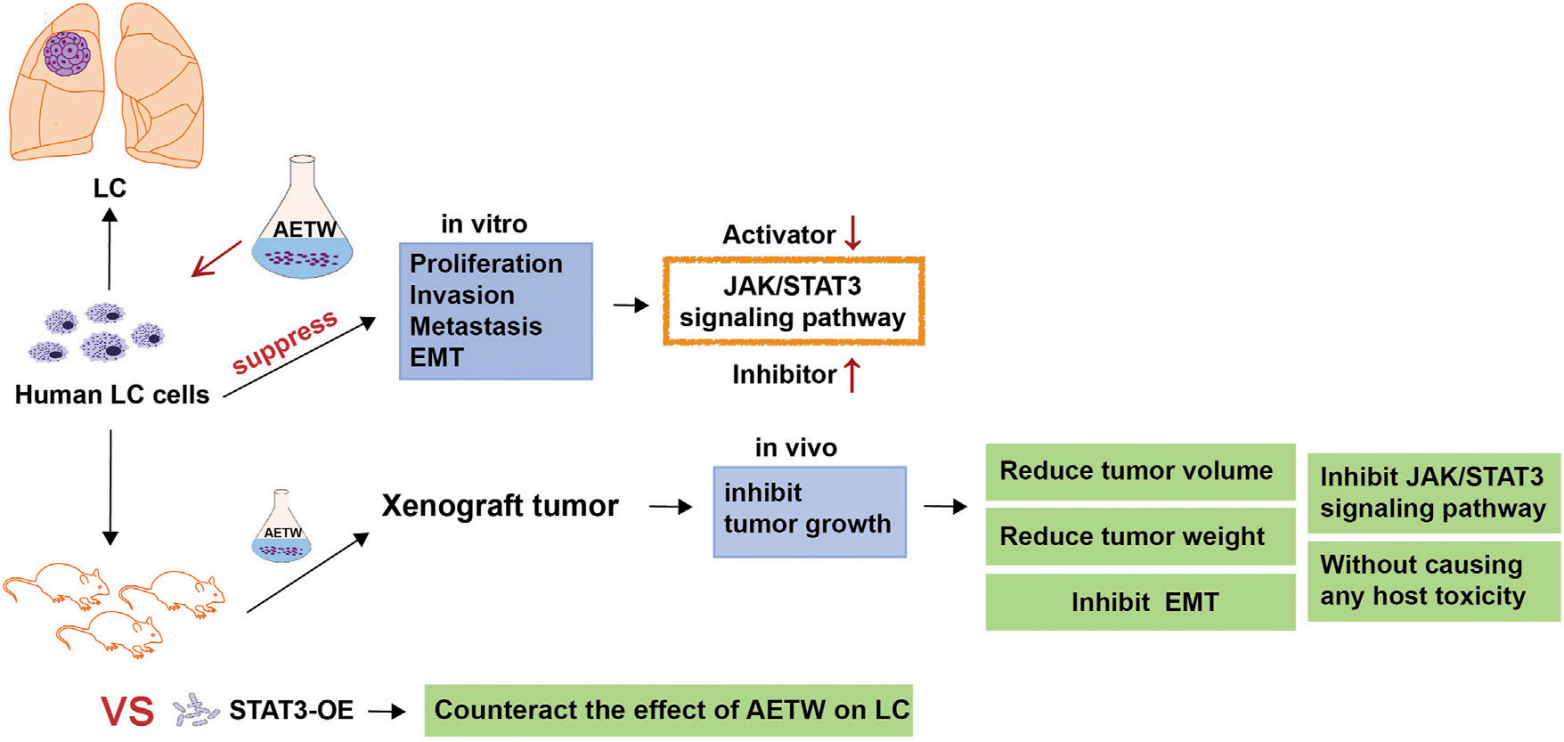
Experimental flowchart.

Invasion and metastasis are complex processes during the development and progression of cancers (Zhang et al., 2021b; Ni et al., 2021; Sun et al., 2021). Initially, a small subset of cancer cells detaches from the primary malignant lesion, and they suffer morphological changes like epithelial‐to‐mesenchymal transition (EMT). Later, they migrate through the extracellular matrix, invade the neighboring or distant tissues, and start the malignant growth, thus resulting in cancer metastasis (Steeg, 2016). Generally, cadherins and vimentin are involved in the EMT process. It is reported that IL-8 secreted by tumor-infiltrating macrophages may promote the EMT and invasiveness of cancer cells by activating the JAK2/STAT3 signaling pathway (Fu et al., 2015). Also, TCM decoction was found to markedly inhibit the EMT of lung cancer cells by downregulating the activator of STAT3 and mesenchymal markers such as N-cadherin and vimentin (Li et al., 2017). The JAK/STAT3 axis is a classic cell signaling pathway mediated by enzyme-linked receptors in cells and participates in the pathogenesis and development of cancers (Bai et al., 2021). It consists of tyrosine kinase–related receptors, tyrosine kinase JAK, and transcription factor STAT. The abnormal activation of the JAK/STAT3 signaling pathway in tumors has been proven associated with malignant phenotypes such as uncontrolled cell proliferation, invasion, and migration (Venugopal et al., 2020). As previously revealed, Taxol extracted from TW took a synergistic role in significantly increasing cytotoxicity and inducing the apoptosis of colorectal carcinoma cells by regulating STAT3 signaling (Yang et al., 2021). Of note, preclinical studies proposed that the combination of JAK inhibitor and Taxol extracted from TW might be conducive to weakening tumor load for inflammatory breast cancer and have given the evidence on showing good tolerance to its clinical activity (Lynce et al., 2021), suggesting tightness between TW and the JAK/STAT3 axis. Our study aims to explore the functional effects of AETW on the growth, migration, and EMT of LC cells and to investigate the involvement of the JAK/STAT3 signaling pathway in this process, which has been shown in Figure 1.

## Materials and Methods

### Materials and Reagents

*Taxus wallichiana* var. *chinensis* (Pilg.) Florin was purchased from the pharmacy in the First Affiliated Hospital of Zhejiang Chinese Medical University (Hangzhou, China), which was manufactured by the Ningbo Taikang Yew Biological Engineering Company (Batch No. 100513). Human lung cancer (LC) cell lines, including A549, NCI-H358, HCC827, and MRC-5, were purchased from the Cell Bank of the Chinese Academy of Sciences (Shanghai, China). They were cultured by 0.25% trypsin digestion in the mediums of F-12K (21127-022, GIBCO, Hangzhou, China), RPMI-1640 (C11875500BT, GIBCO, Hangzhou, China), and MEM (41500034, GIBCO, Hangzhou, China) for 1–2 mins at 37°C in a humidified atmosphere consisting of 5% CO_2_ separately, where NCI-H358 and HCC827 were both cultured in RPMI-1640 medium. The antibodies included N-cadherin (1:3000, ab98952, Abcam), E-cadherin (1:2000, 20874-1-AP, Proteintech), vimentin (1:2000, ab92547, Abcam), p-JAK (1:500, ab32101, Abcam), p-STAT3 (1:500, ab76315, Abcam), JAK (1:1000, 3230, CST), STAT3 (1:1000, 06-596, Merck), and GAPDH (1:5000, 70-Mab5465-040, MultiSciences).

### Preparation of AETW

The preparation of AETW was based on the procedure detailed in a previous study (Zhang et al., 2021a). A total of 12 g of TW was put into a clean porcelain jar and soaked overnight. Eight times of water was added into the container and boiled. The liquid medicine was poured out until it was cooled down. The operation was repeated twice. All the liquid was gathered and filtered by a vacuum filter and then concentrated to 48 mg/ ml in the induction cooker. Finally, the liquid medicine was, respectively, diluted to 0.125, 0.25, 0.5, 1.0, 2.0, and 4.0 mg/ ml and stored at 4°C.

### UPLC-Q/TOF-MS/MS Detection

The 2777C UPLC system (Waters, US) equipped with an ACQUITY UPLC BEH C18 column (150 mm × 2.1 mm, 1.7 μm, Waters, US) was used for the chromatographic separation of AETW. The mobile phase was composed of A (0.1% formic acid + acetonitrile) and B (0.1% formic acid solution), the flow rate was 0.3 ml/ min, and the injection volume of each sample was 1 μL. The gradient elution conditions were as follows: 0∼4.5 mins, 99%–85% B; 4.5∼12 mins, 85%∼75% B; 12∼15 mins, 75%∼65% B; 15∼16 mins, 65%∼60% B; 16∼20 mins, 60%∼20% B; and 20∼22 mins, 20% B.

A Q/TOF mass spectrometer (MS) coupled with a turbo ion spray and the positive and negative ion modes was employed for analyzing the active medicinal constituent. The optimized parameter settings were as follows: source temperature of 600°C (ESI+ and ESI-) and ion spray voltage floating of 5500 V/-4500 V. The MS scan range was from 50 to 1,500 Da, and the production scan range was from 25 to 1,000 Da. The second-order mass spectrum was acquired by IDA coupled with high sensitivity. The specialized parameters were set up including the declustering potential of ±60 V (ESI + and ESI-) and the collision energy of 35 ± 15 eV. During the course of data collection, the screening was completed based on the exact mass number of compounds from AETW, isotope distribution ratio, and SCIEX OS software configured with TCM MS/MS Library.

### Lung Cancer Cell Strain Screening

The lung cancer cell strain was examined as described previously (Fang et al., 2018). Four types of human LC cell lines were measured by CCK-8 (Dojindo, Japan), as per the instruction. In short, the cells were seeded in 96-well plates at a density of 5 × 10^3^ cells per well and mixed with AETW at various concentrations (0, 0.125, 0.25, 0.5, 1.0, 2.0, 4.0 mg/ ml) for 24, 48, and 72 h. After each well was added with 10 μL CCK-8 solution and incubated at 37°C in the dark for 1 h, the OD value and the IC_50_ index were obtained by GraphPad Prism v5 software. The strains with better responses were selected for subsequent experiments.

### Detection of Stable STAT3-OE Cell Strains

The overexpressed STAT3 (STAT3-OE) lentivirus and the control ones (STAT3-NC) were purchased from VigeneBio (Shandong, China) and performed based on the manufacturer’s instructions. The cells were seeded in six-well plates at a density of 1 × 10^5^ cells per well. Until the cells adhered to the wall, they were divided into two groups and transfected by STAT3-OE and STAT3-NC lentivirus for 48 h, respectively. Before the STAT3 expression level of two groups was detected by western blot, puromycin (puro) at the concentration of 2.5 μg/ ml was added for the selection of stable cell lines. The cell viabilities of stable strains were measured by CCK-8 (Dojindo, Japan) in the above method (Fang et al., 2018), where the mediums were mixed with AETW at 0.5 mg/ml and only cultured for 48 h.

### Anti-Tumor Activity Assay *In Vitro*

#### Cell Proliferation Assay

The anti-proliferation assay was performed as described previously (Wang et al., 2017a; Chen et al., 2019). The two cell lines with better responses were planted in growth culture medium with starvation treatment. After being performed with AETW at different concentrations (0, 0.125, 0.25, 0.5 mg/ ml) for 48 h, they were collected, washed with PBS, and centrifuged. Resuspended cells were mixed with 1 ml DNA staining solution and 10 μL permeabilization solution and then incubated in the dark for 30 min. At last, flow cytometry was applied to the analysis of cell proliferation.

#### Wound Healing Assays

The wound healing assay was performed by cell migration as described previously (Wang et al., 2020a). The un-transduced LC cells were cultured into six-well plates until the degree of integration exceeded 90%. A 200 µL pipette tip was used to create three straight “scratches” marked “0h.” Then, the cells were maintained in the mediums containing AETW at gradient concentrations (0, 0.125, 0.25, 0.5 mg/ ml) for 24 h, and the scratch width was measured during the process. The stable cell lines infected by two kinds of lentivirus were conducted in the same method again, where the mediums were mixed with AETW at 0.5 mg/ml.

#### Transwell Assays

The transwell assay was detected as mentioned previously (Zheng et al., 2018). The un-transduced LC cells were performed with AETW at four levels of concentrations. Then, the cells were planted into 24-well transwell chambers and cultured in mediums containing 20% serum. After incubation for 12 h, the cells on the upper surface were wiped off with cotton swabs, and the cells on the lower surface were fixed with 1% crystal violet for 20 min and washed with PBS three times. The stained cells were counted by an inverted microscope. The same measurement was conducted on the cells infected by lentivirus again, where the mediums were mixed with AETW at 0.5 mg/ml.

#### Confocal Microscopic Analysis for Cadherin and Vimentin Expressions

The sample for confocal microscopic analysis was made as mentioned previously (Zhou et al., 2018). The un-transduced LC cells were kept in AETW at different concentrations and washed with PBS throughout the following process. After fixing with 4% paraformaldehyde for 20 mins, rupturing with 0.1% Triton X-100 for 20 mins, and blocking with 1% BSA for 1 h, the membranes were incubated with the related primary antibodies overnight and secondary antibody in the dark, respectively, followed by the addition of 10 ng/ ml DAPI at 4°C. The cells were observed through a fluorescence microscope and confocal microscope.

### Anti-Tumor Activity Assay *In Vivo*

#### Xenograft Tumor in Nude Mice

The xenograft model was established as previously described (Chen et al., 2019). Male nude mice purchased from SLAC (Shanghai, China) were divided into four groups randomly and kept under pathogen-free condition of 20–25°C, RH 40–70%. A549, A549-STAT3-OE, and A549-STAT3-NC xenografts models of male nude mice were constructed by subcutaneous injections of 5.0 × 10^6^ cells, whereas the first and the second groups were both injected with A549. The mice were all exposed to AETW except the first group. The tumor size and animal body weights were measured twice a week as previously described (Chen et al., 2019). Five weeks later, the mice were sacrificed and the tumors were removed and measured for calculation. To observe the pathomorphological changes better, the tumor tissue fixed in 4% formaldehyde solution was embedded in paraffin and cut into pieces. After dewaxing and rehydrating with xylene and ethanol, the slides were stained with hematoxylin and eosin. Finally, the slides were sealed with neutral gum.

#### Immunohistochemistry

The immunohistochemistry assay was performed as described previously (Zheng et al., 2018). The embedded tumor tissue was cut into 4-m sections, which were dewaxed and rehydrated subsequently and then cultured with 3% hydrogen peroxide for 15 min and boiled with citrate buffer (pH 6.0); the cooled slides were incubated with primary antibodies overnight at 4°C and mixed with secondary antibodies after being transferred to room temperature. The slides were washed with PBS when each step mentioned above was completed. Finally, they were stained with the DAB substrate and counterstained with hematoxylin. Each experiment was conducted in triplicate.

### Western Blot Analysis

The western blot assay was performed as described previously (Zhang et al., 2015; Wang et al., 2017a). Two types of cells were processed by mediums that met experimental requirements and washed with PBS as preceding steps. The cell lysates were separated by SDS and transferred onto the PVDF membrane. The membrane was blocked with 5% skim milk in TBST at room temperature for an hour and then probed with the primary antibodies at 4°C overnight, followed by incubation with the corresponding secondary antibody at room temperature for an hour. Eventually, the membrane was washed three times with TBST and examined by chemiluminescence. For tumor tissues, protein samples were prepared by standard methods, and the protein concentration was determined by BCA. Subsequent steps from electrophoresis with polyacrylamide gel to chemiluminescence development with ECL followed the cell western blot above. Hence, the protein expressions of N-cadherin, E-cadherin, vimentin, p-JAK, JAK, p-STAT3, and STAT3 *in vivo* in tumor tissue could be obtained. All experiments were repeated in triplicate.

### Statistical Analysis

Each set of experiment was repeated in triplicate. The results were presented as mean ± SD. Comparisons between groups were assessed with one-way analysis and Student’s t-test by using SPSS 20.0 statistical software. The difference was considered statistically significant if *p <* 0.05, dramatically significant if *p <* 0.01, and not significant if *p >* 0.05.

## Results

### Mass Spectrometric Analysis of AETW

**FIGURE 2:**
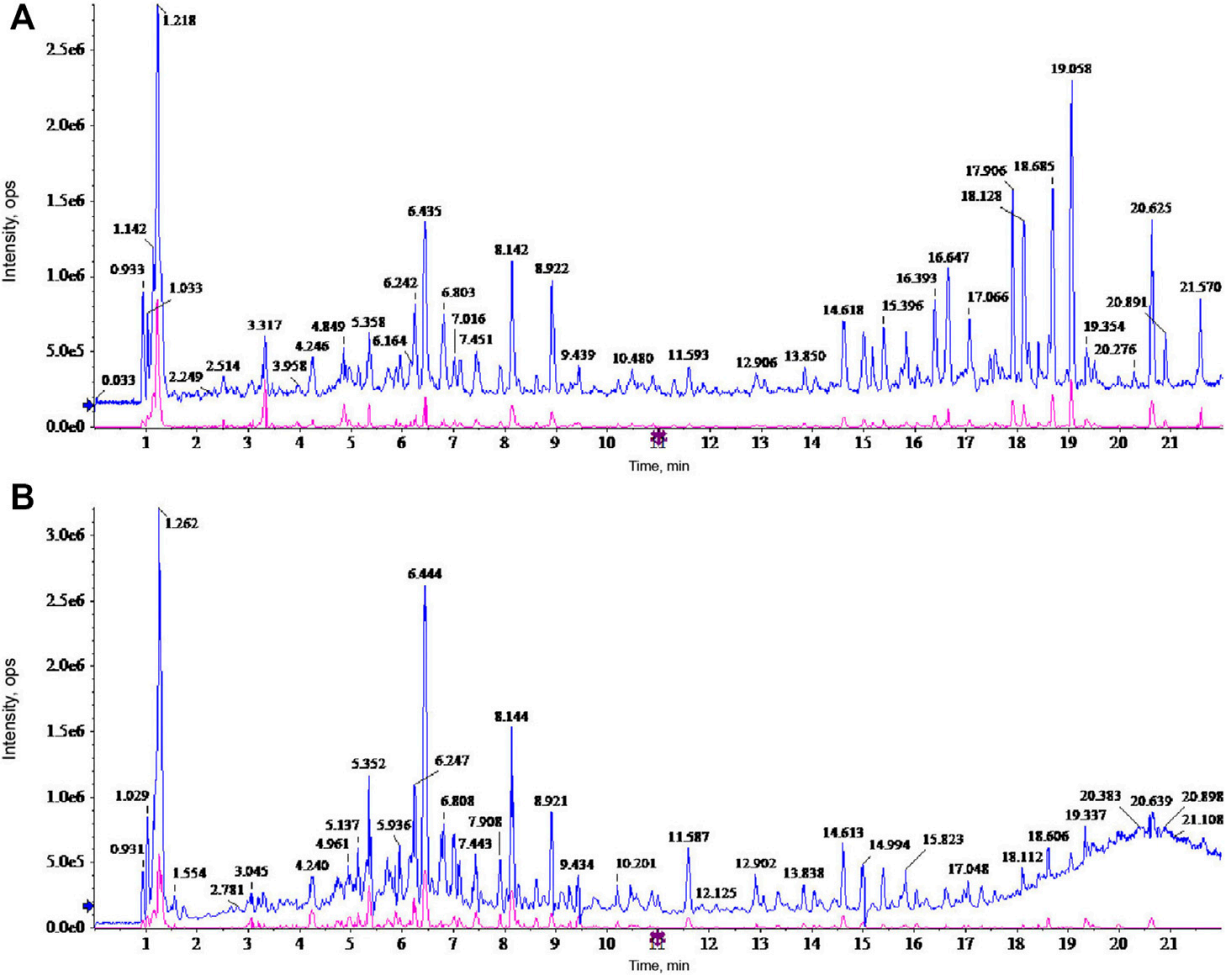
Ion current chromatograms of AETW in positive **(A)** and negative **(B)** modes, respectively.

**FIGURE 3:**
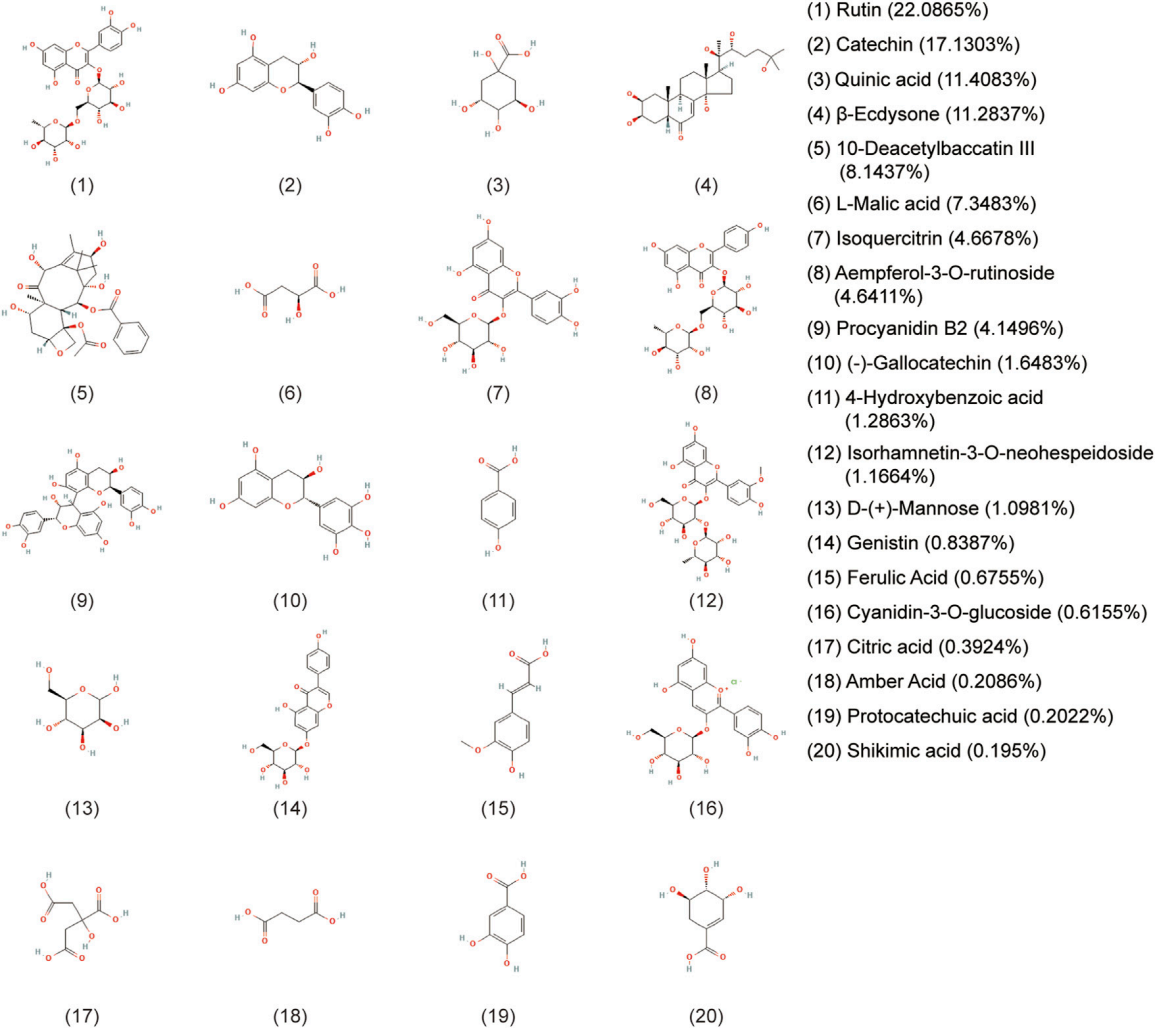
Molecular structural formulas of the major chemical components in AETW and their ratios.

A holistic approach was adopted to identify the specific chemical compounds in AETW. Both positive and negative ion modes were conducted for a mass spectra coverage of the constituents of AETW. However, the negative ion mode was chosen for the standardization of the extract due to more acidic substances in AETW. The total ion current chromatograms of AETW and the major chemical components are presented in Figures 2, 3, respectively.

### AETW Inhibited the Proliferation of LC Cells by Arresting Cell Cycle Progression

**FIGURE 4:**
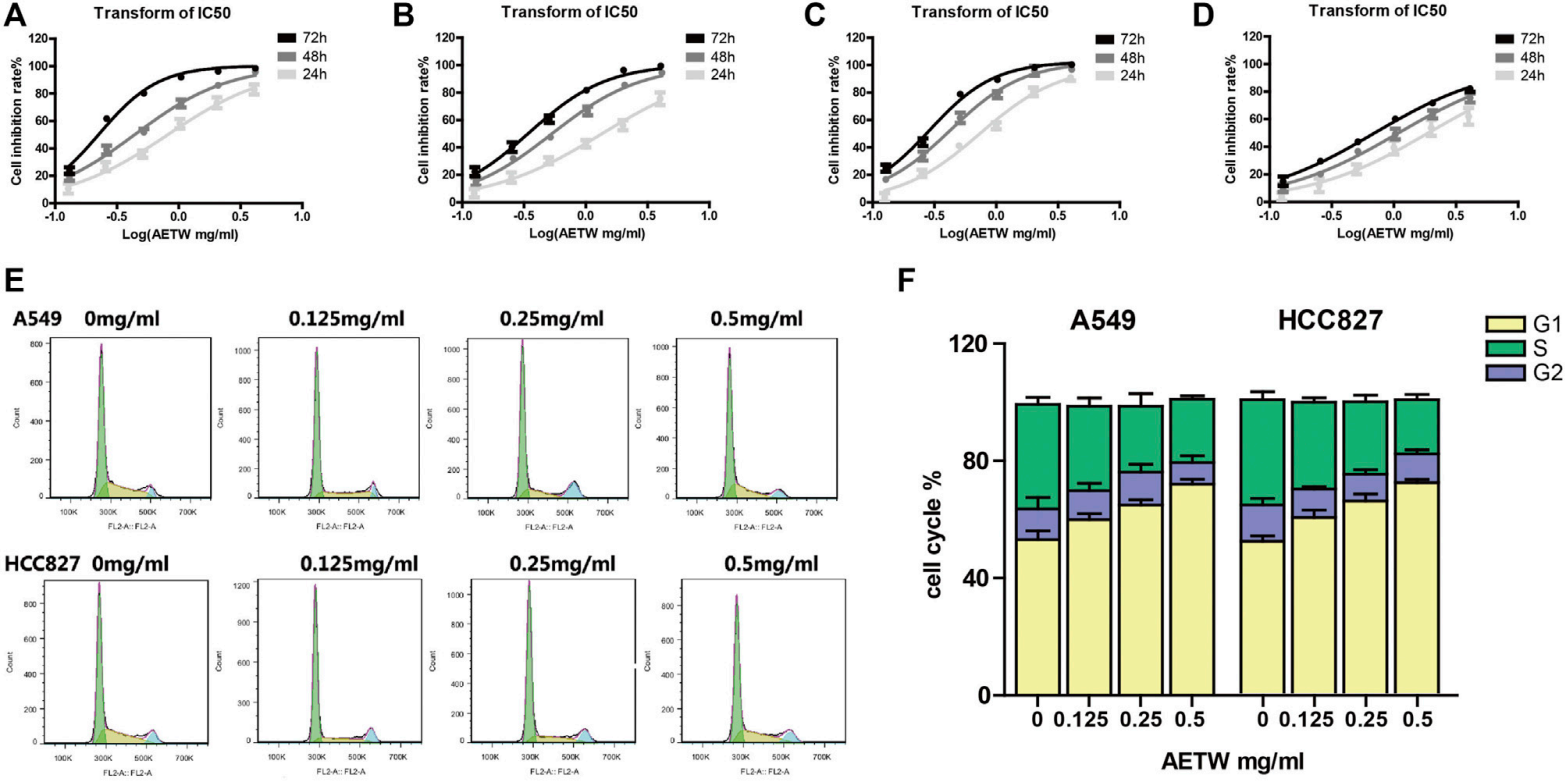
*In vitro* cytotoxicity screening of LC (lung cancer) cells for AETW. The effects on A549 **(A)** and HCC827 **(C)** cells were more obvious than on NCI-H358 **(B)** cells and MRC-5 **(D)** cells. The result of LC cell cycle arrest was induced by AETW. Flow cytometry analyses illustrated that cells accumulated in S and G2 phases **(E)**. Quantitative analyses of cell cycle distributions of A549 and HCC827 cells **(F)**.

As shown in Figures 4A–D and Table 1, AETW inhibited the growth of all LC cell lines with IC_50_ values ranging from 0.74 to 1.89 μg/ ml at 24 h, 0.42 to 1.06 μg/ ml at 48 h, and 0.22 to 0.68 μg/ ml at 72 h. The IC_50_ value of the MRC-5 lung cell line was higher than that in other three cell lines, especially at 72 h, indicating that AETW could selectively act on LC cells with little effect on normal lung cells.

**TABLE 1:** IC_50_ index of A549, NCI-H358, HCC827, and MRC-5 cells with AETW treatment for 24, 48, and 72 h.

Time	IC_50_ (mg/ml)			
	A549	NCI-H358	HCC827	MRC-5
24 h	0.78	1.35	0.74	1.89
48 h	0.43	0.51	0.42	1.06
72 h	0.22	0.34	0.28	0.68

To investigate whether the arrested cell cycle progression mainly attributes to this inhibitory effect, the flow cytometry assay was conducted for analyzing cell cycle distribution of AETW-induced LC cells. It is shown that AETW concentration-dependently arrested the A549 and HCC827 cells’ cycle in the G1 phase (Figures 4E,F).

### AETW Suppressed the Migration and Invasion Abilities of LC Cells

**FIGURE 5:**
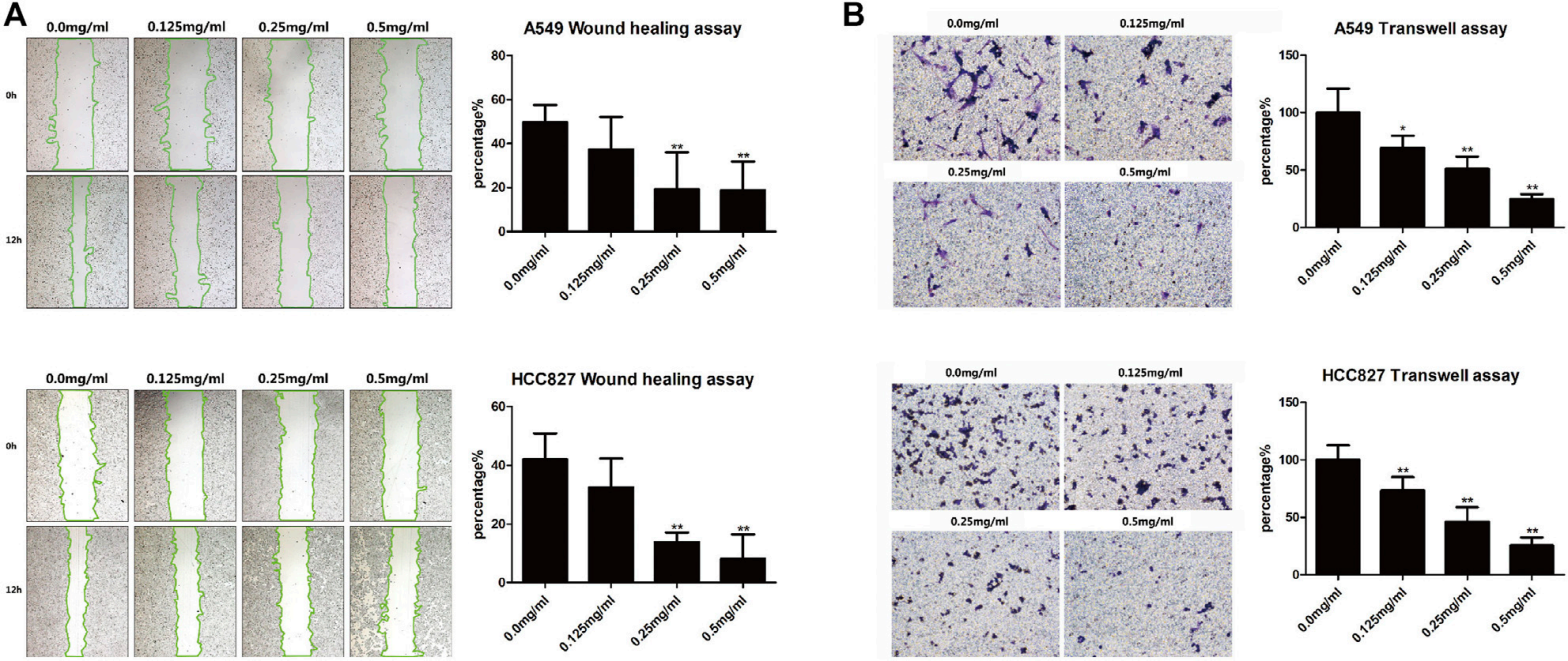
AETW suppressed the migration and invasion of LC cells with increasing concentrations and exposure time. **(A)** Detections of cell migration by the wound healing assay and their quantitative analyses. **(B)** Detections of cell invasion using transwell plates and their quantitative analyses. Compared with the first group, *p < 0.05, **p < 0.01.

The *in vitro* wound healing assay and transwell assay were performed to assess migration and invasion capabilities of LC cells, respectively. After AETW exposure, the changes of A549 and HCC827 cells revealed that the wound healing rate of cells was on the decline (Figure 5A). Subsequently, we detected the invasion capability of LC cells with the transwell assay. As shown in Figure 5B, the invasion percentage was concentration-dependently inhibited by AETW in both A549 and HCC827 cells.

### AETW Downregulated EMT and Inactivated the JAK/STAT3 Signaling Pathway in LC Cells

**FIGURE 6:**
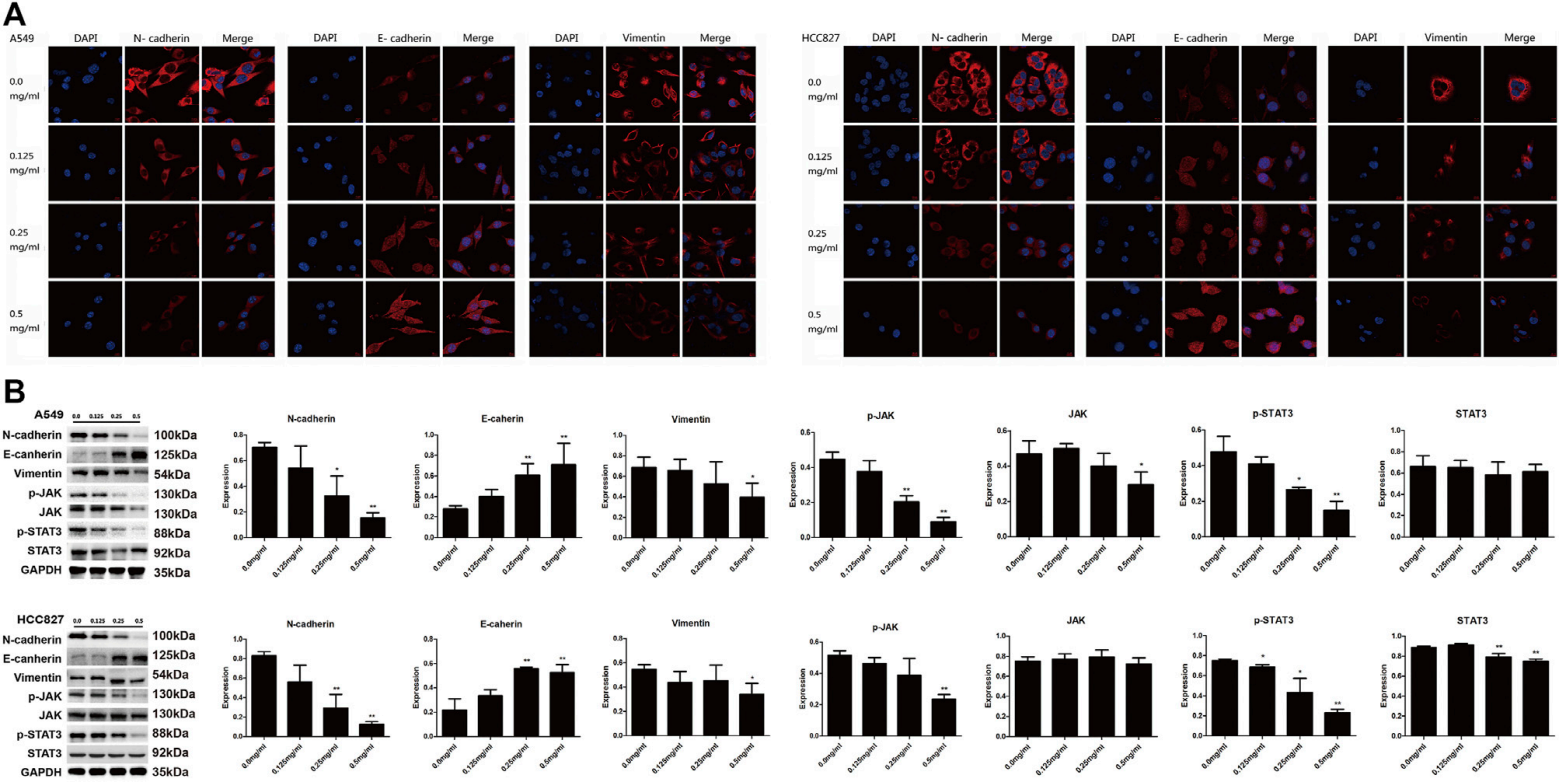
AETW decreased the expressions of EMT markers and JAK/STAT3 signaling regulators. Confocal microscope inspections **(A)** and immunoblot analyses **(B)** of the expressions of cadherins, vimentin, and JAK/STAT3 axis signaling proteins using the corresponding antibodies. Compared with the first group, *p < 0.05, **p < 0.01.

As a crucial process in cancer development, the EMT contributes to strengthening of metastatic capacity of LC cells (Syn et al., 2016; Mashouri et al., 2019). We therefore investigated expression levels of EMT-related markers with western blot and confocal microscopic analyses. As shown in Figures 6A,B, N-cadherin and vimentin were both downregulated, while E-cadherin was upregulated by AETW in a concentration-dependent manner. Moreover, we determined expression levels of key factors in the JAK/STAT3 signaling pathway. Our results illustrated that AETW markedly reduced the phosphorylation expression levels of JAK and STAT3 in concentration-dependent and time-dependent manners, suggesting that the JAK/STAT3 signaling pathway may be mainly responsible for AETW-regulated alleviation of LC progression.

### Overexpression of STAT3 Counteracted the Effects of AETW on LC *In Vivo* and *In Vitro*

**FIGURE 7:**
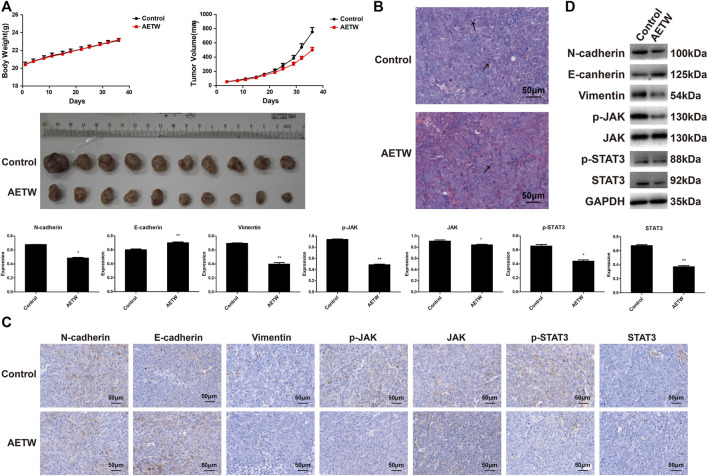
AETW inhibited LC cell growth and metastatic ability *in vivo*. **(A)** Measurements of tumor volume and mice body weight. **(B)** Hematoxylin–eosin staining results of tumor tissue. **(C)** Immunohistochemistry analyses of EMT markers and JAK/STAT3 signaling regulators. **(D)** Western blot analyses of expressions of cadherins, vimentin, and JAK/STAT3 signaling proteins and their quantitative analyses. Compared with the control group, *p < 0.05, **p < 0.01.

To further verify the therapeutic value of AETW *in vivo*, we generated a subcutaneous LC model in nude mice. As displayed in Figure 7A and Tables 2, 3, the tumor volume and body weight of AETW-treated mice were significantly larger and higher than those without drug intervention. Moreover, the tumor suppression rate was 33.43% (Table 4), implying the satisfactory therapeutic effect of AETW.

**TABLE 2:** Mice body weight of the two experiment groups (control, AETW).

Group	Number (n)		Body weight (g)	
	D1	D35	D1	D35
Control	10	10	20.43 ± 0.80	23.11 ± 0.71
AETW	10	10	20.40 ± 0.38	23.19 ± 0.36

There is no significance between the two groups.

**TABLE 3:** Tumor volume from mice of the two experiment groups (control, AETW).

Group	Tumor volume (mm^3^)
Control	753 ± 196
AETW	500 ± 115^**^

Compared with the control group, **p < 0.01.

**TABLE 4:** Tumor weight from mice of the two experiment groups (control, AETW).

Group	Tumor weight (g)	IR (%)
Control	0.6533 ± 0.1689	−
AETW	0.4349 ± 0.1202^**^	33.43

Compared with the control group, **p < 0.01.

Hematoxylin and eosin (H&E) staining illustrated the pathological changes of LC tissues in mice. Similarly, we found that the number of mitotic nuclei of AETW group was notably reduced than that of controls. Immunohistochemistry and western blot were conducted to evaluate the *in vivo* metastatic ability of LC in nude mice, and the results were consistent with *in vitro* findings (Figures 7B–D).

**FIGURE 8:**
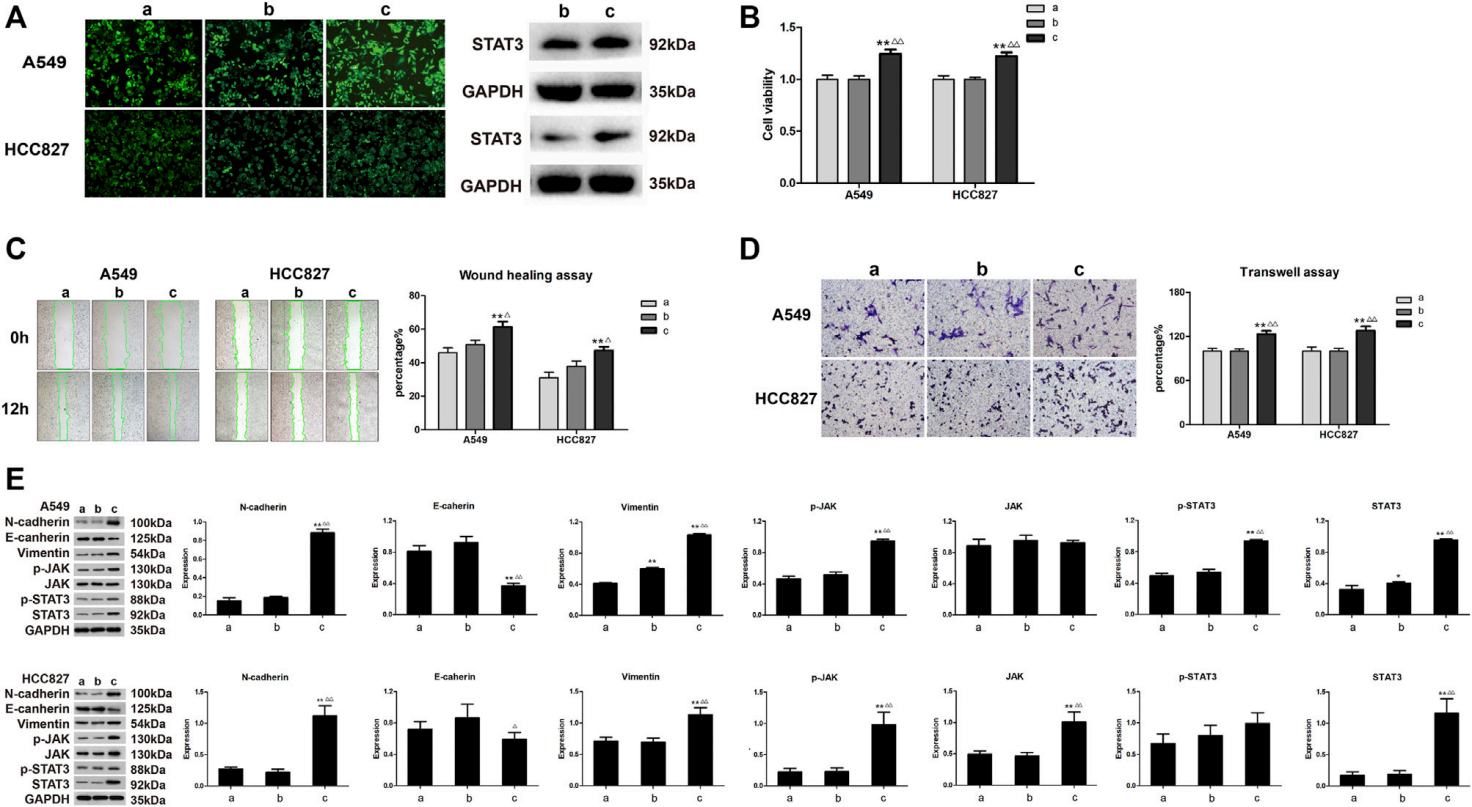
The effects of STAT3-OE for A549 and HCC827 cells on LC cells were opposite to those being performed with AETW. **(A)** Expressions of stable transduced cell lines. **(B)** Quantitative analyses of A549 and HCC827 cell viabilities by the CCK-8 assay. **(C)** Detections of migration of LC cells treated with STAT3-OE by the wound healing assay and their quantitative analyses. **(D)** Detections of invasion of LC cells treated with STAT3-OE using transwell plates and their quantitative analyses. **(E)** Expressions of cadherins, vimentin, and JAK/STAT3 axis signaling proteins by western blot analyses and their quantitative analyses. Compared with the control group, *p < 0.05, **p < 0.01. Compared with the STAT3-NC + AETW group, ^△^ *p <* 0.05, ^△△^ *p <* 0.01. a: control group; b: STAT3-NC + AETW; c: STAT3-OE + AETW.

To examine whether AETW-induced downregulation of STAT3 was the reason to cause the inhibition of proliferation and invasion of LC cells, the cells stably transfected by STAT3-OE lentivirus participated in the follow-up experiments (Figure 8A). As suggested in Figure 8B, the intervention of overexpressed STAT3 significantly accelerated the viabilities of A549 cells and HCC827 cells. Meanwhile, cell migration and invasion capacities and EMT were all increasing and the JAK/STAT3 signaling pathway was more active in the STAT3-OE group (Figures 8C–E).

**FIGURE 9:**
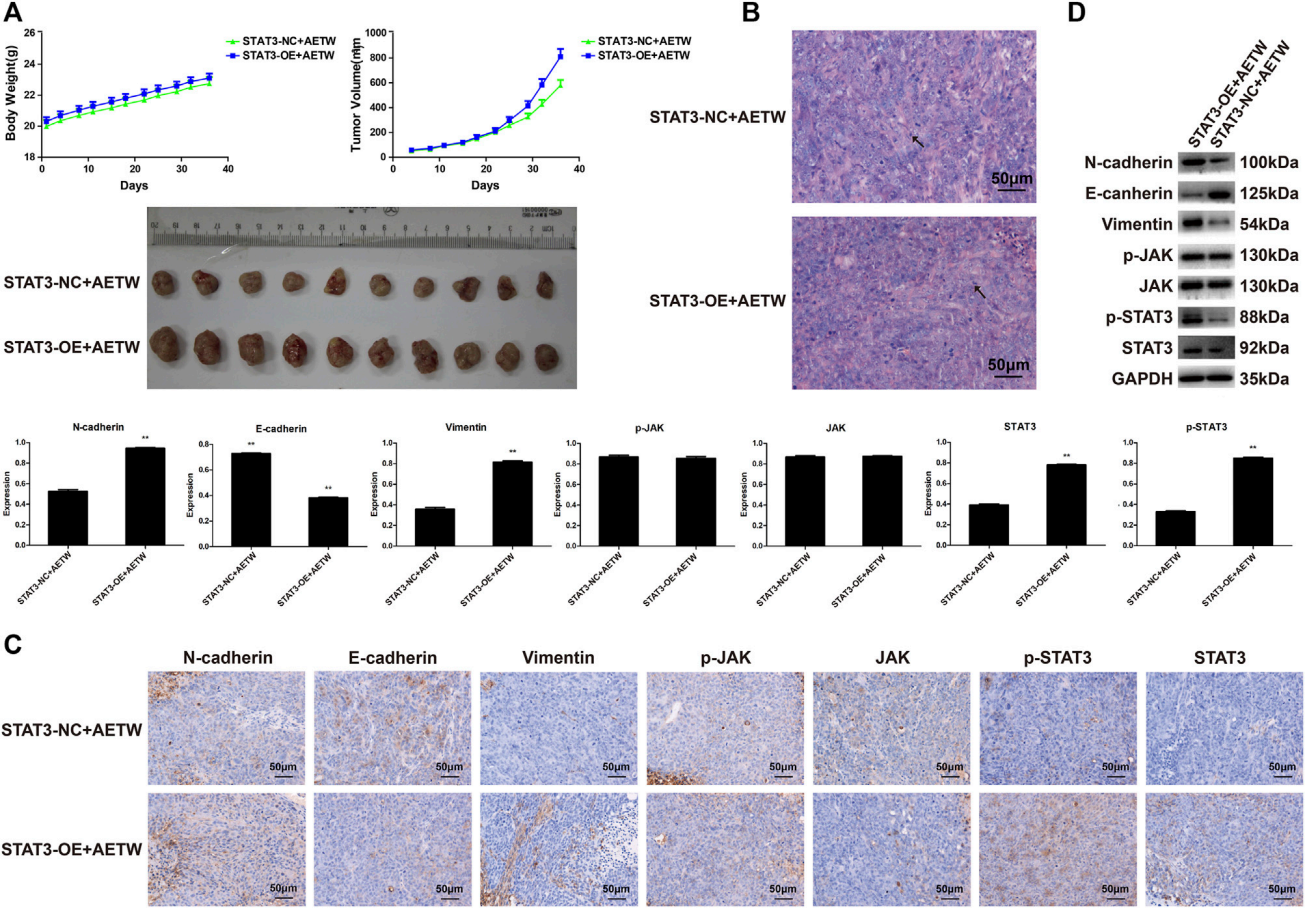
STAT3 overexpression reversed the effect of AETW *in vivo* on LC tissue. **(A)** Measurements of tumor volume and mice body weight. **(B)** Hematoxylin–eosin staining results of tumor tissue. **(C)** Immunohistochemistry analyses of EMT markers and JAK/STAT3 signaling regulators. **(D)** Western blot analyses of expressions of cadherins, vimentin, and JAK/STAT3 signaling proteins and their quantitative analyses. Compared with the STAT3-NC + AETW group, **p < 0.01.

Then, we designed the xenograft tumor model in nude mice using the above-mentioned method. As shown in Tables 5, 6, the body weight and tumor volume detected in the experimental group were higher and larger than those in the STAT3-NC + AETW group. Notably, the tumor suppression rate was -52.65%, suggesting the effect of AETW was counteracted by the addition of overexpressed STAT3 (Table 7). Meanwhile, it could not only accelerate the tumor growth but also promote the EMT and upregulate the expressions of active forms of JAK and STAT3 (Figures 9A–D).

**TABLE 5:** Mice body weight of the two experiment groups (STAT3-NC + AETW, STAT3-OE + AETW).

Group	Number (n)		Body weight (g)	
	D1	D35	D1	D35
STAT3-NC + AETW	10	10	19.99 ± 1.31	22.75 ± 1.34
STAT3-OE + AETW	10	10	20.31 ± 0.89	23.11 ± 0.91

There is no significance between the two groups.

**TABLE 6:** Tumor volume from mice of the two experiment groups (STAT3-NC + AETW, STAT3-OE + AETW).

Group	Tumor volume (mm^3^)
STAT3-NC + AETW	579 ± 140
STAT3-OE + AETW	811 ± 199^**^

Compared with the STAT3-NC + AETW group, **p < 0.01.

**TABLE 7:** Tumor weight from mice of the two experiment groups (STAT3-NC + AETW, STAT3-OE + AETW).

Group	Tumor weight (g)	IR (%)
STAT3-NC + AETW	0.4656 ± 0.1342	−
STAT3-OE + AETW	0.7107 ± 0.1763^**^	−52.65

Compared with the STAT3-NC + AETW group, **p < 0.01.

Collectively, we proposed that AETW at least partially contributed to the inhibition of proliferation and metastasis of LC cells by regulating the JAK/STAT3 signaling pathway.

## Discussion

TCM has become a widely concerned topic due to the potential anti-tumor properties of its components. TW has been recognized as the anticancer plant due to its attractive source of Taxol and possession of more than 14 chemical ingredients with the anti-tumor property (Han, 1994). As generally considered, the etiology of LC is due to smoking, dietary habits, decreased immunity, and genetic factors (Park et al., 2021; Yuan et al., 2021). In TCM theory, the accumulation of cancerous toxin is the leading cause of LC. Professor Zhou Zhong-ying highlighted the core position of cancerous toxin and claimed that cancerous toxin obstructing the lung should be put forward as an independent syndrome differentiation and treatment (Cai et al., 2020). TW exerts the detoxication function, which is exactly corresponding to the pathogenesis of LC. Therefore, it is necessary to explore the underlying mechanism of the anti-tumor effect of TW.

After identifying the chemical compositions and their ratio in AETW, our findings showed that AETW suppressed the growth of LC cells in time-dependent and concentration-dependent manners. Among the tested LC cell lines, A549 and HCC827 cells presented the most pronounced response to AETW treatment, which thereby were selected in the following experiments. Compared to those treated with AETW at a lower concentration, the highest arrested rate in A549 cells and HCC827 cells treated with 0.5 mg/ ml AETW was detected in the G1 phase (71.8–73.7% *vs*. 59.9–62.4% and 65.0–67.9%), indicating that AETW could concentration-dependently arrest the LC cell cycle in the G1 phase. In addition, the *in vitro* wound healing assay and transwell assay suggested that AETW could weaken cell migration and invasion capabilities of LC cells in a concentration-dependent manner.

The EMT is a well-known molecular mechanism associated with cancer metastasis, which is characterized by the loss of epithelial phenotypes and the acquisition of mesenchymal phenotypes (Babaei et al., 2021). N-cadherin and vimentin are the early- and late-stage biomarkers of EMT, respectively (Jiang et al., 2019). To acquire an invasive feature, tumor cells are transformed from the normal epithelium expressing downregulated E-cadherin to motile mesenchymal cancer cells highly expressing the adhesion molecule N‐cadherin, which is in a process known as cadherin switching (Zhou et al., 2015; Rosso et al., 2017). Furthermore, vimentin, serving as a cytoskeletal protein that stabilizes the nucleus and organelles, interacts directly with transcription factors that function primarily in the nucleus to activate EMT-related transcription genes (Liu et al., 2017; Gong et al., 2019). Satelli et al. (Satelli and Li, 2011) suggested that vimentin was overexpressed in many malignant tumor tissues or cancer cell lines of epithelial origin like liver cancer, lung cancer, etc. As a crucial signaling molecule and transcription factor, vimentin participates in the rapid growth, infiltration, metastasis, and apoptosis of tumor cells. Consequently, the results of western blot showed downregulated N-cadherin and vimentin and upregulated E-cadherin in LC cells treated with AETW, suggesting that AETW significantly inhibited the invasion and metastasis of LC.

The JAK/STAT3 pathway has been broadly described as a vital signaling pathway involved in cancer cell biology (Ran et al., 2021). The fundamental role of STAT3 in cell proliferation has been previously reported (Chen et al., 2021). A comprehensive meta-analysis reported that the overexpression of STAT3 was correlated with low disease-free survival and poor prognosis of the stomach, lung, brain, liver, bone, prostate, and pancreas cancers (Wu et al., 2016). In gastric carcinoma, cancer cell–secreted TGF-β1 interacted with IL-6, thus inducing STAT3 activation and promoting the extensive metastasis of cancer cells (Wang et al., 2020b). Through targeting STAT3, miRNA-130b is regarded as a prognostic marker to suppress proliferation and induce apoptosis of pancreatic cancer cells (Zhao et al., 2013). Moreover, STAT3 is specifically associated with the promoter of HOXD-AS1 and then activates HOXD-AS1 transcription, resulting in a poor prognosis and advanced stage of lymph node metastasis in hepatocellular carcinoma patients (Wang et al., 2017b). Despite manifold consequences of excessive activation of STAT3 in cancer cells, STAT3 is responsible for accelerating proliferation, apoptosis resistance, angiogenesis, and metastasis (Bromberg et al., 1999; Verhoeven et al., 2020).

In the experiment with the addition of overexpressed STAT3, the inhibitory effect on malignant phenotypes of LC cells was counterbalanced, and there was a decline in the expressions of phosphorylated JAK and STAT3, the active form of JAK and STAT3, suggesting that AETW might exert the inhibitory effect by inactivating the JAK/STAT3 signaling pathway. As a vital upstream protein of STAT3, Janus kinase (JAK) is the most relevant biological molecule to catalyze STAT3 activation. Due to the stimulation of chronic cytokines, the receptor-related JAK is phosphorylated, which further recruits and phosphorylates STAT3. A formed dimer is then translocated into the nucleus and binds to the specific DNA in the gene promoter region to activate the transcription of downstream target genes (Timofeeva et al., 2012; Huynh et al., 2017). Hu et al. (2016) reported that, in non-small-cell lung cancer treated with Momelotinib, a JAK inhibitor, combined with the EGFR inhibitor cetuximab, the anticancer activity of cetuximab on its drug-resistant tumors has been greatly improved. Recent studies have uncovered that driven by the tumor microenvironment rich in IL-6 family cytokines, the JAK pathway activates the contractility of actin in tumor cells, induces the remodeling of extracellular matrix, and creates a favorable environment for the collective migration of tumor cells (Lokau et al., 2019). Strikingly, actomyosin contractility itself positively modulates the activity of the transcription factor STAT3 that is located downstream of JAK1, demonstrating the positive feedback within the signaling network (Sanz-Moreno et al., 2011). It has been proven that the STAT3 protein is hardly to be specifically targeted in clinical research mainly because of the high homology with the other seven STAT proteins (Huynh et al., 2017). Because of the regulatory effects on both p-JAK and p-STAT3 expressions, our present study indicated that AETW might be a promising drug as a JAK activation inhibitor used in the targeted treatment of LC.

An aqueous extract of AETW’s shows selective cytotoxicity towards LC cells but it has very poor bioavailability affecting the embodiment of actual clinical efficacy (Jin et al., 2013; Liu et al., 2015). Many factors influence the poor bioavailability of AETW including poor solubility in aqueous solutions and administration method (Liu et al., 2015). Interestingly, nano delivery of bioactive compounds makes contribution to enhancing bioavailability and biodistribution through the improvement of uptake, stability, and solubility (Zare et al., 2021). The technology has been utilized in a variety of chemicals including paclitaxel (Crintea et al., 2021), which may provide a basis for the clinical application of AETW.

## Conclusion

In summary, we revealed that the aqueous extract of *Taxus wallichiana* var. *chinensis* (Pilg.) *Florin* could suppress the proliferation, migration, and invasion capabilities of LC cells and the protein expression of the EMT and the JAK/STAT3 signaling pathway. Meanwhile, our findings indicated that the active form of JAK and STAT3 was most sensitive to the effects but their expressions rose again when LC cells were transfected by STAT3 overexpression. As a future research direction, it is essential to go deep into the detailed molecular mechanisms and investigate the clinical efficacy and side effect of AETW as a JAK activation inhibitor used in the targeted treatment of LC.

## Data Availability

The original contributions presented in the study are included in the article/Supplementary Material, and further inquiries can be directed to the corresponding authors.

## Abbreviations

AETW, the aqueous extract of *Taxus wallichiana* var. *chinensis* (Pilg.) Florin; LC, lung cancer; OE, overexpression; TCM, traditional Chinese medicine; TW, *Taxus wallichiana* var. *chinensis* (Pilg.) Florin.
