# Supplementary material for: Taxus wallichiana var. chinensis (Pilg.) Florin Aqueous Extract Suppresses the Proliferation and Metastasis in Lung Carcinoma via JAK/STAT3 Signaling Pathway
Source: Front Pharmacol. 2021 Nov 16;12:736442. doi: 10.3389/fphar.2021.736442 (PMC8635059; doi:10.3389/fphar.2021.736442)
Supplement: Supplementary file 1 [file Table1.DOCX]

| Mode | Component Name | Area (cps) | Ratio  (%) | Retention Time (min) | Formula | Precursor Mass | Found  At Mass | Mass Error (ppm) | Library Score | Isotope Ratio Difference |
| --- | --- | --- | --- | --- | --- | --- | --- | --- | --- | --- |
| Positive | Betaine | 105000 | 1.9987 | 1.15 | C5H11NO2 | 118.086 | 118.0863 | 0.1 | 100 | 0.7 |
|  | Proline | 80090 | 1.5245 | 1.22 | C5H9NO2 | 116.071 | 116.0706 | -0.3 | 98.5 | 2.7 |
|  | Cytidine | 23250 | 0.4426 | 1.28 | C9H13N3O5 | 244.093 | 244.0929 | 0.3 | 98.7 | 1.6 |
|  | Adenine | 82070 | 1.5622 | 1.28 | C5H5N5 | 136.062 | 136.0618 | 0.4 | 96.1 | 0.2 |
|  | Vitamin B6 | 5806 | 0.1105 | 1.32 | C8H11NO3 | 170.081 | 170.0814 | 1.2 | 93.1 | 1.9 |
|  | Adenosine | 282900 | 5.3851 | 2.51 | C10H13N5O4 | 268.104 | 268.104 | -0.3 | 98 | 5.8 |
|  | Guanosine | 14300 | 0.2722 | 2.65 | C10H13N5O5 | 284.099 | 284.099 | 0.3 | 100 | 1.8 |
|  | Phenylalanine | 31270 | 0.5952 | 3.46 | C9H11NO2 | 166.086 | 166.0864 | 0.8 | 99.8 | 1.5 |
|  | (-)-Gallocatechin | 37310 | 0.7102 | 3.86 | C15H14O7 | 307.081 | 307.0814 | 0.6 | 98.5 | 1.8 |
|  | Procyanidin B2 | 242200 | 4.6103 | 4.82 | C30H26O12 | 579.15 | 579.1496 | -0.2 | 96.4 | 11.2 |
|  | Epicatechin | 864200 | 16.4502 | 5.36 | C15H14O6 | 291.086 | 291.0862 | -0.3 | 95.4 | 3.8 |
|  | Eriodictyol | 21870 | 0.4163 | 5.94 | C15H12O6 | 289.071 | 289.0709 | 0.9 | 76.5 | 4.5 |
|  | Ginkgolide B | 24050 | 0.4578 | 5.96 | C20H24O10 | 425.144 | 425.1425 | -4 | 75.6 | 5.3 |
|  | Anisaldehyde | 65500 | 1.2468 | 6.16 | C8H8O2 | 137.06 | 137.0598 | 0.7 | 77.4 | 1.6 |
|  | Vitamin B2 | 9904 | 0.1885 | 6.25 | C17H20N4O6 | 377.146 | 377.1457 | 0.3 | 93.8 | 3.2 |
|  | Rutin | 1736000 | 33.0451 | 8.14 | C27H30O16 | 611.161 | 611.1607 | 0.1 | 97.8 | 8.5 |
|  | Quercetin | 214200 | 4.0773 | 8.15 | C15H10O7 | 303.05 | 303.0501 | 0.5 | 82 | 1.6 |
|  | Isoquercitrin | 131900 | 2.5107 | 8.62 | C21H20O12 | 465.103 | 465.1029 | 0.3 | 99.6 | 8 |
|  | β-Ecdysone | 722300 | 13.7491 | 8.92 | C27H44O7 | 481.316 | 481.3158 | -0.3 | 86.7 | 8.9 |
|  | Aempferol-3-O-rutinoside | 376500 | 7.1667 | 9.44 | C27H30O15 | 595.166 | 595.166 | 0.4 | 98 | 10.9 |
|  | Isorhamnetin-3-O-neohespeidoside | 55660 | 1.0595 | 9.74 | C28H32O16 | 625.176 | 625.1766 | 0.5 | 100 | 9.6 |
|  | Cyanidin-3-O-glucoside | 36100 | 0.6872 | 10.41 | C21H20O11 | 449.108 | 449.108 | 0.4 | 93.4 | 1.1 |
|  | Genistin | 66350 | 1.2630 | 10.52 | C21H20O10 | 433.113 | 433.1129 | 0 | 99.5 | 7.5 |
|  | Apigenin | 7122 | 0.1356 | 15.94 | C15H10O5 | 271.06 | 271.0598 | -1.2 | 88.4 | 5.5 |
|  | Cephalomannine | 17580 | 0.3346 | 19.41 | C45H53NO14 | 832.354 | 832.354 | 0.2 | 89 | 14.6 |
| Negative | D-(+)-Mannose | 185000 | 1.0981 | 1.13 | C6H12O6 | 179.056 | 179.0561 | -0.1 | 92.7 | 2.5 |
|  | Glutamic acid | 23290 | 0.1382 | 1.13 | C5H9NO4 | 146.046 | 146.046 | 0.7 | 98.5 | 1.6 |
|  | Quinic acid | 1922000 | 11.4083 | 1.23 | C7H12O6 | 191.056 | 191.056 | -0.8 | 87.9 | 1.4 |
|  | L-Malic acid | 1238000 | 7.3483 | 1.31 | C4H6O5 | 133.014 | 133.0141 | -0.8 | 79.4 | 1.7 |
|  | Shikimic acid | 32860 | 0.1950 | 1.54 | C7H10O5 | 173.046 | 173.0454 | -0.6 | 84.5 | 2.2 |
|  | Citric acid | 66110 | 0.3924 | 2.03 | C6H8O7 | 191.02 | 191.0197 | -0.4 | 98.8 | 2 |
|  | Amber Acid | 35140 | 0.2086 | 2.38 | C4H6O4 | 117.019 | 117.0194 | 0.4 | 90.7 | 1.1 |
|  | Guanosine | 26980 | 0.1601 | 2.65 | C10H13N5O5 | 282.084 | 282.0843 | -0.3 | 95.1 | 5.5 |
|  | 4-Hydroxybenzoic acid | 216700 | 1.2863 | 3.2 | C7H6O3 | 137.024 | 137.0243 | -0.8 | 94.3 | 3.5 |
|  | Phenprobamate | 15710 | 0.0932 | 3.46 | C9H11NO2 | 164.072 | 164.0718 | 0.4 | 88.7 | 2 |
|  | (-)-Gallocatechin | 277700 | 1.6483 | 3.86 | C15H14O7 | 305.067 | 305.0665 | -0.7 | 84.1 | 7.3 |
|  | Protocatechuic acid | 34070 | 0.2022 | 4.08 | C7H6O4 | 153.019 | 153.0194 | 0.3 | 95.6 | 2.3 |
|  | L-Tryptophan | 9463 | 0.0562 | 4.66 | C11H12N2O2 | 203.083 | 203.0824 | -0.8 | 84.3 | 1.5 |
|  | Procyanidin B2 | 699100 | 4.1496 | 4.82 | C30H26O12 | 577.135 | 577.1344 | -1.3 | 96 | 14.9 |
|  | Catechin | 2886000 | 17.1303 | 5.36 | C15H14O6 | 289.072 | 289.0714 | -1.2 | 93.9 | 8.3 |
|  | Caffeic acid | 3999 | 0.0237 | 5.93 | C9H8O4 | 179.035 | 179.0352 | 1.1 | 75.9 | 4.2 |
|  | Rutin | 3721000 | 22.0865 | 8.14 | C27H30O16 | 609.146 | 609.145 | -1.8 | 98.4 | 12.9 |
|  | Pinoresinol Diglucoside +HCOOH | 2914 | 0.0173 | 8.45 | C32H42O16.HCOOH | 727.245 | 727.2442 | -1.8 | 94.7 | 5.3 |
|  | Isoquercitrin | 786400 | 4.6678 | 8.62 | C21H20O12 | 463.088 | 463.088 | -0.5 | 96.5 | 8 |
|  | Ferulic Acid | 113800 | 0.6755 | 8.89 | C10H10O4 | 193.051 | 193.0505 | -0.9 | 90.3 | 4.9 |
|  | β-Ecdysone +HCOOH | 1901000 | 11.2837 | 8.92 | C27H44O7.HCOOH | 525.307 | 525.3065 | -0.8 | 97.8 | 12.3 |
|  | Aempferol-3-O-rutinoside | 781900 | 4.6411 | 9.44 | C27H30O15 | 593.151 | 593.1502 | -1.7 | 99 | 9.7 |
|  | Isorhamnetin-3-O-neohespeidoside | 196500 | 1.1664 | 9.74 | C28H32O16 | 623.162 | 623.1611 | -1 | 96 | 13.7 |
|  | Cyanidin-3-O-glucoside | 103700 | 0.6155 | 9.96 | C21H20O11 | 447.093 | 447.0928 | -1 | 88.1 | 11.3 |
|  | Genistin | 141300 | 0.8387 | 10.52 | C21H20O10 | 431.098 | 431.0979 | -1.1 | 98.9 | 9.1 |
|  | Luteolin | 7386 | 0.0438 | 14.14 | C15H10O6 | 285.04 | 285.0404 | -0.2 | 95.9 | 3.7 |
|  | Quercetin | 18730 | 0.1112 | 14.16 | C15H10O7 | 301.035 | 301.0352 | -0.7 | 86.8 | 2.7 |
|  | 10-Deacetylbaccatin III +HCOOH | 1372000 | 8.1437 | 14.61 | C29H36O10.HCOOH | 589.229 | 589.2286 | -0.8 | 73.9 | 14.4 |
|  | Apigenin | 12530 | 0.0744 | 15.94 | C15H10O5 | 269.046 | 269.0453 | -0.9 | 93.3 | 2.9 |
|  | Amentoflavone | 10180 | 0.0604 | 17.58 | C30H18O10 | 537.083 | 537.0824 | -0.6 | 90.1 | 3.2 |
|  | Paclitaxel +HCOOH | 5902 | 0.0350 | 19.55 | C47H51NO14HCOOH | 898.329 | 898.3284 | -0.8 | 95.2 | 12.9 |

**Supplementary Table 1.** Identification of chemical compounds in AETC.
