# Supplementary material for: Taxus wallichiana var. chinensis (Pilg.) Florin Aqueous Extract Suppresses the Proliferation and Metastasis in Lung Carcinoma via JAK/STAT3 Signaling Pathway
Source: Front Pharmacol. 2021 Nov 16;12:736442. doi: 10.3389/fphar.2021.736442 (PMC8635059; doi:10.3389/fphar.2021.736442)

**Uncut original image**

**Western blots of Figure 6**

**A549** N-cadherin


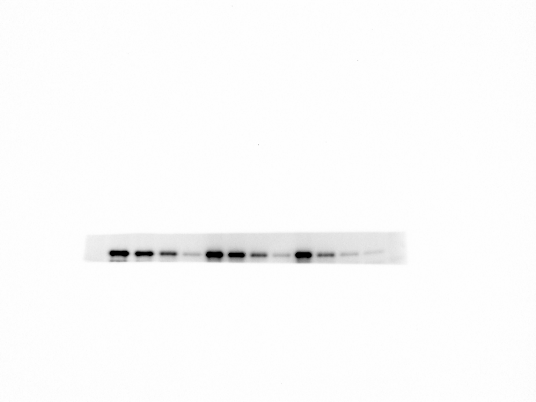


**A549** E-cadherin


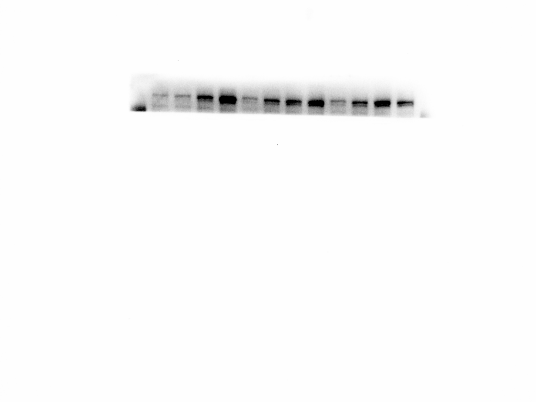


**A549** Vimentin


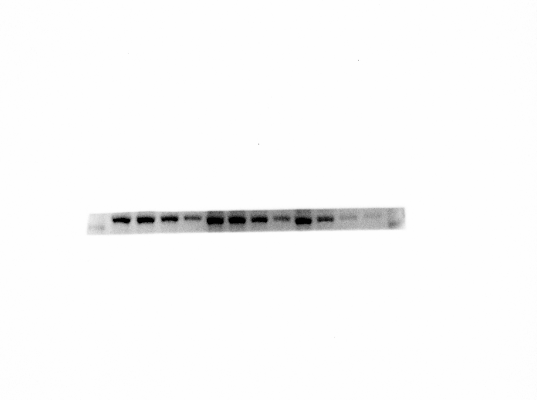


**A549** p-JAK


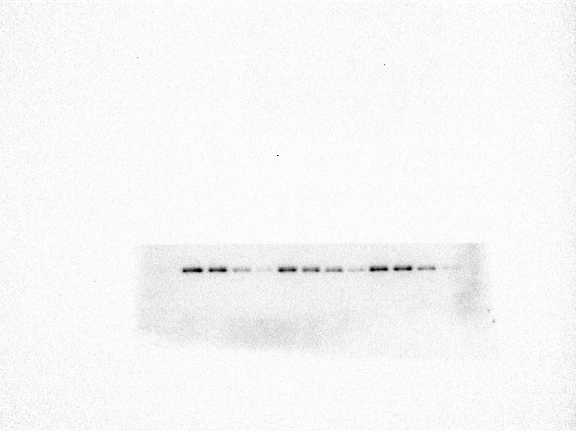


**A549** JAK


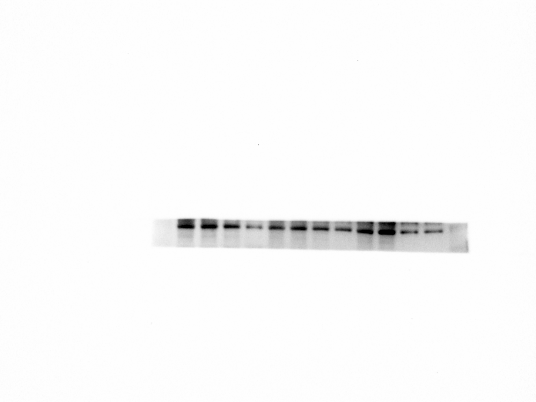


**A549** p-STAT3


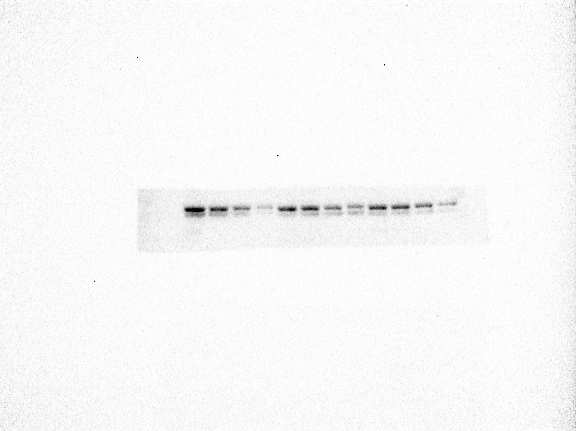


**A549** STAT3


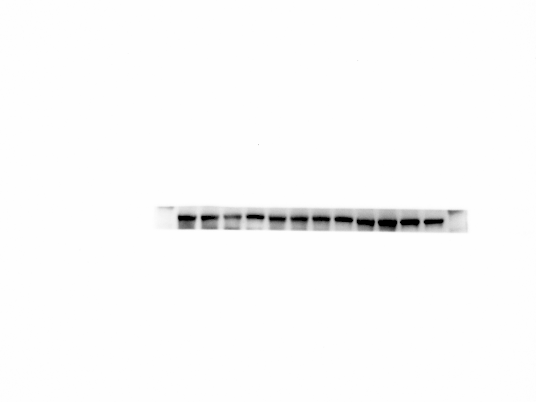


**A549** GAPDH


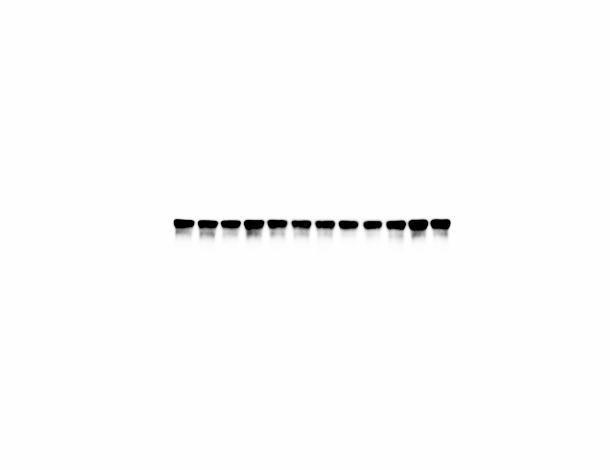


**HCC827** N-cadherin


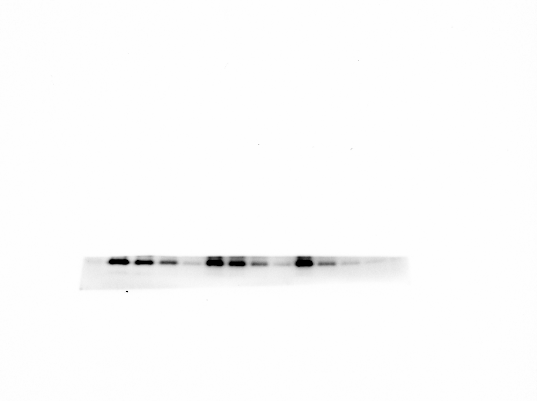


**HCC827** E-cadherin


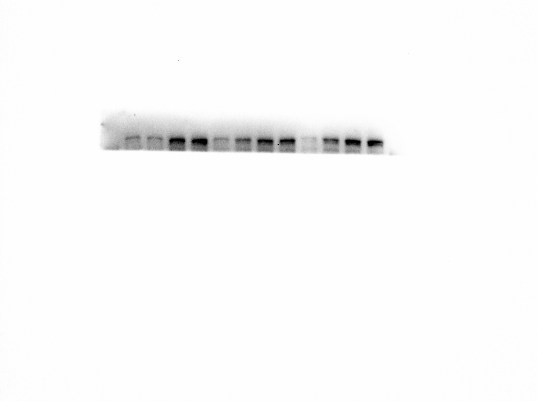


**HCC827** Vimentin


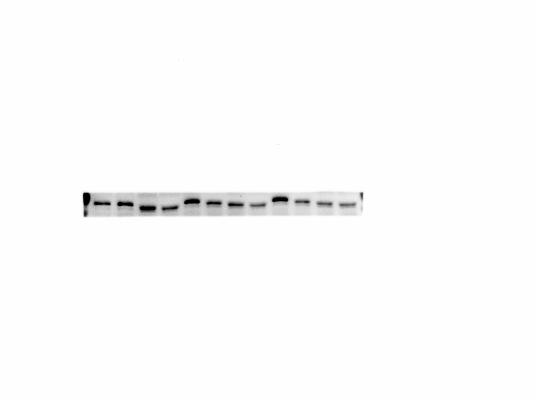


**HCC827** p-JAK


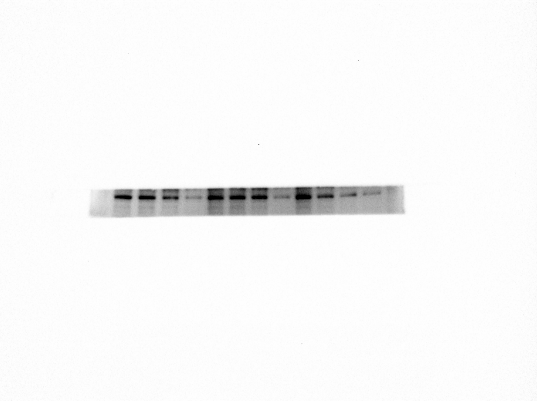


**HCC827** JAK


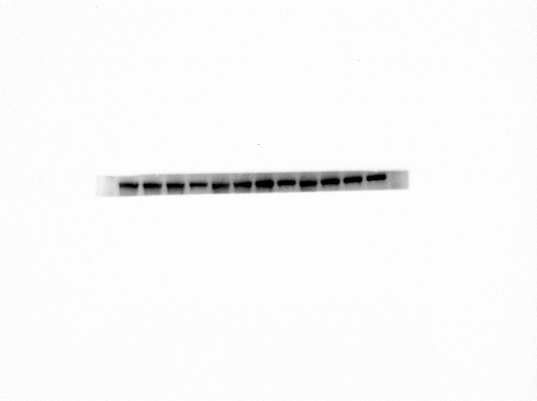


**HCC827** p-STAT3


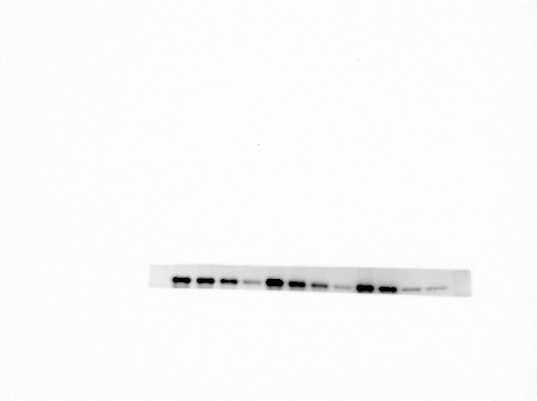


**HCC827** STAT3


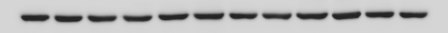


**HCC827** GAPDH


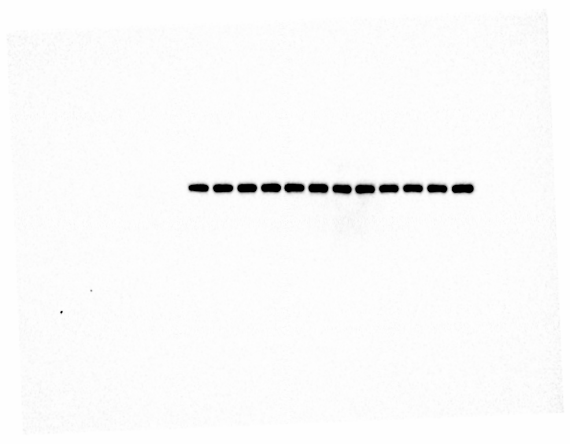


**Western bolts of Figure 7 and 9**

**A549** N-cadherin


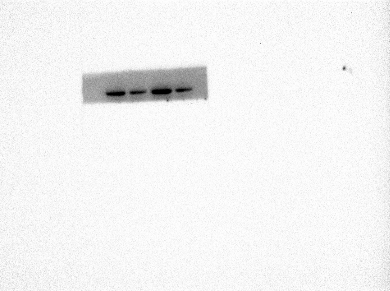


**A549** E-cadherin


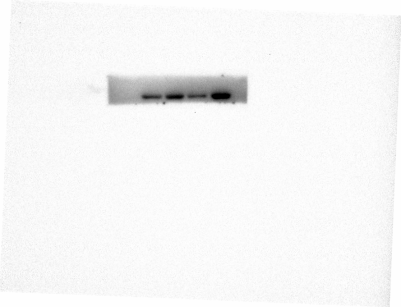


**A549** Vimentin


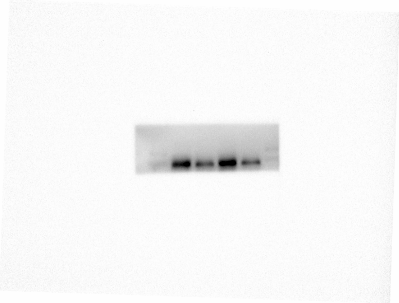


**A549** p-JAK


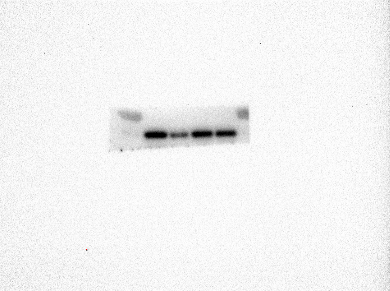


**A549** JAK


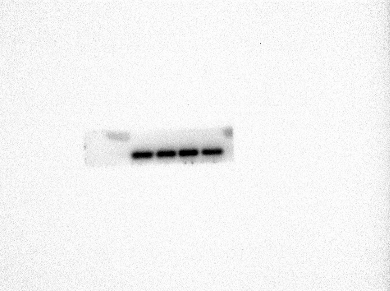


**A549** p-STAT3


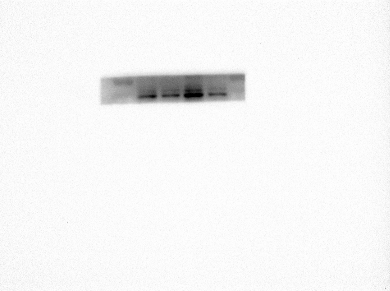


**A549** STAT3


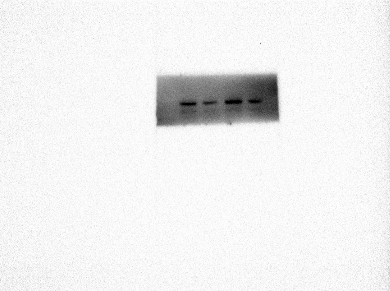


**A549** GAPDH


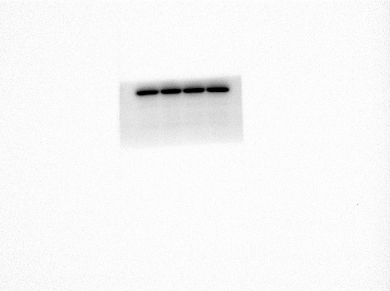


**Western blots of Figure 8**

**A549** N-cadherin


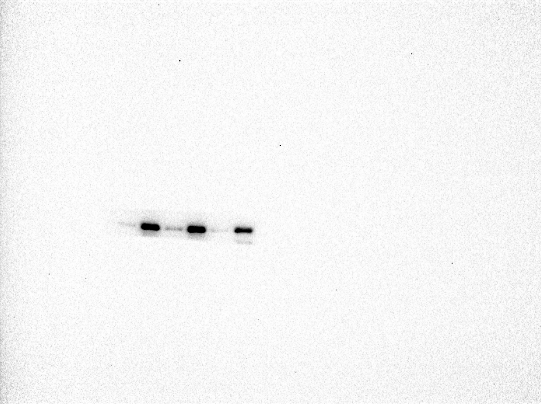


**A549** E-cadherin


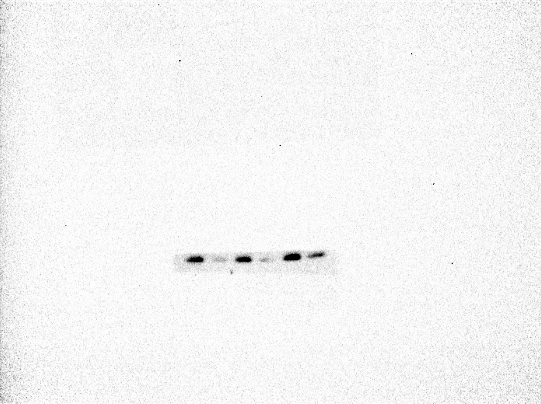


**A549** Vimentin


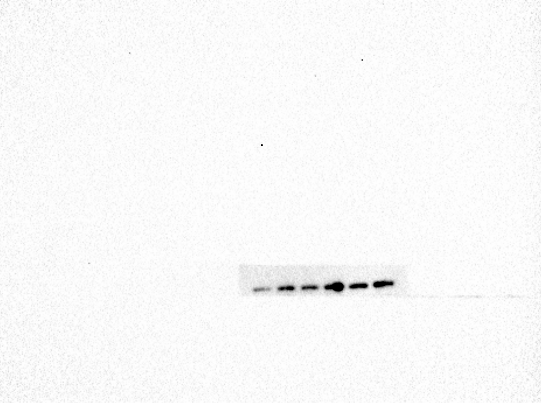


**A549** p-JAK


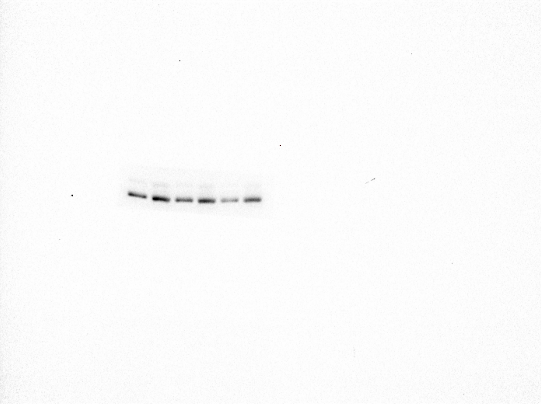


**A549** JAK


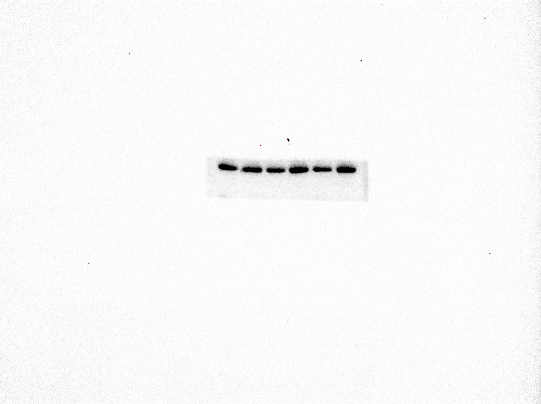


**A549** p-STAT3


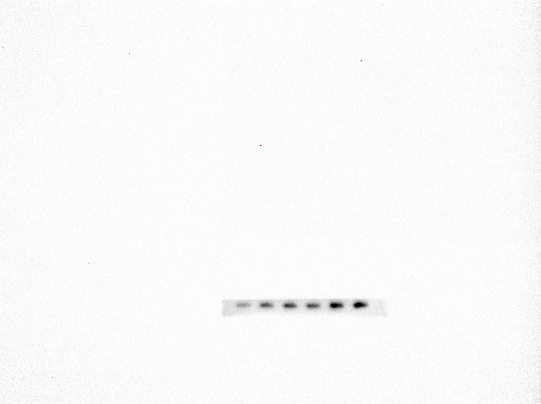


**A549** STAT3


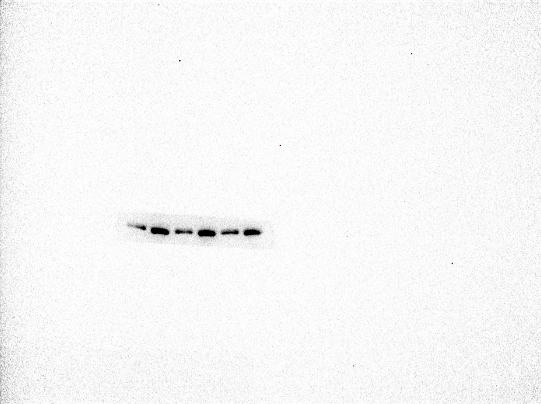


**A549** GAPDH


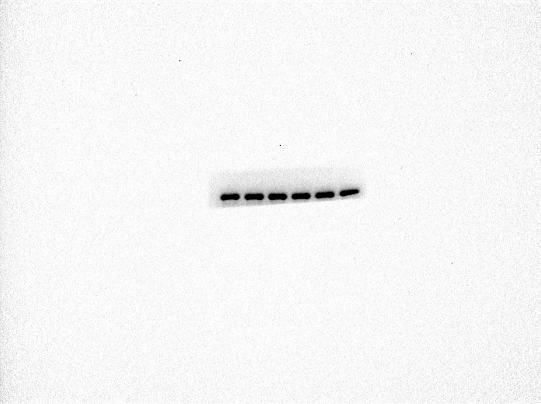


**HCC827** N-cadherin


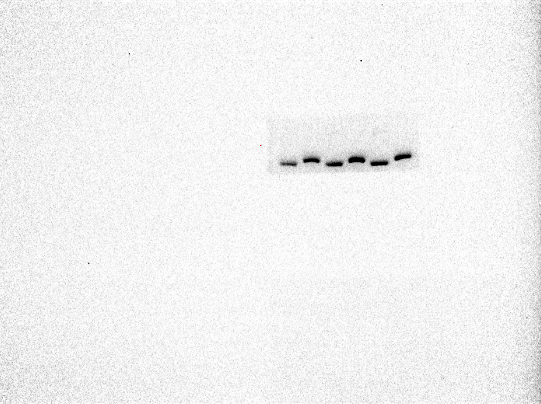


**HCC827** E-canherin


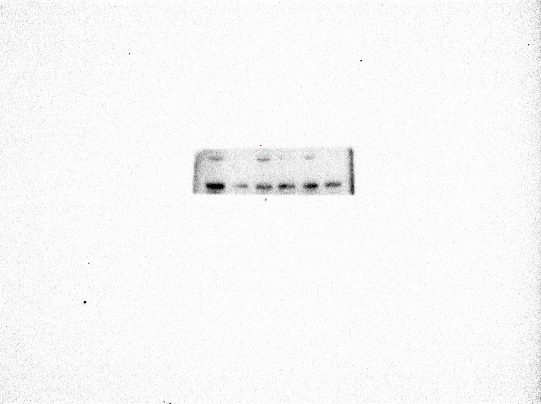


**HCC827** Vimentin


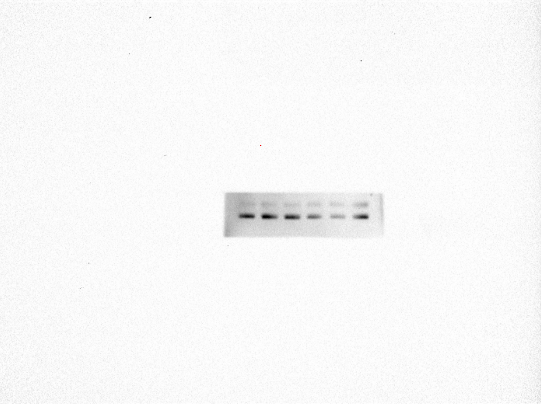


**HCC827** p-JAK


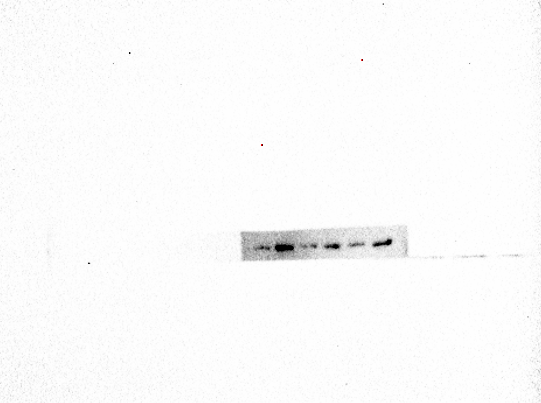


**HCC827** JAK


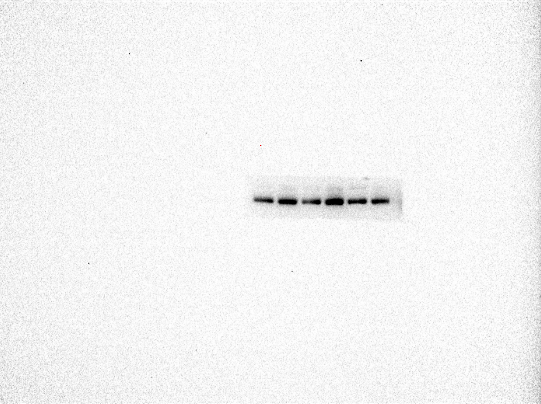


**HCC827** p-STAT3


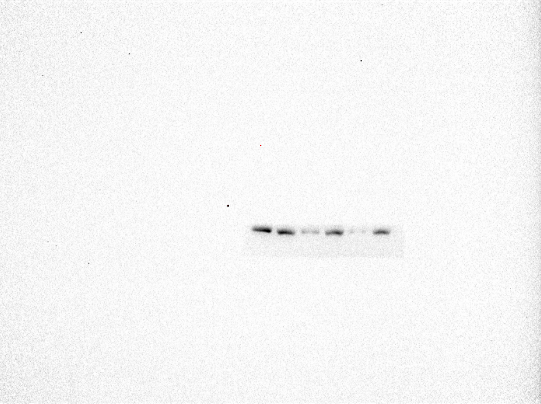


**HCC827** STAT3


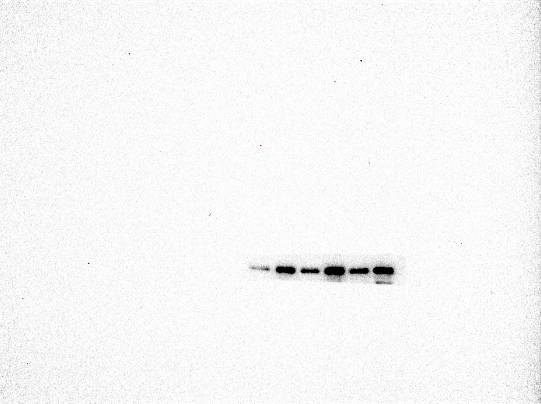


**HCC827** GAPDH


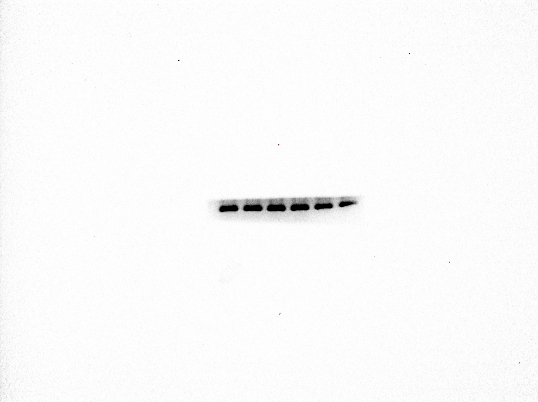

Supplement: Supplementary file 2 [file DataSheet1.ZIP › wb-unedited.docx]
